# Cutaneous squamous cell carcinomas of the nose in a child treated with external radiation therapy: a case report

**DOI:** 10.3332/ecancer.2023.1540

**Published:** 2023-04-28

**Authors:** Samia Khalfi, Zineb El Ayachi, El Mehdi Sadiki, Noureddine Slassi, Wissal Hassani, Fatima Zahra Farhane, Zenab Alami, Touria Bouhafa

**Affiliations:** 1 Department of Radiation Therapy, Oncology Hospital, HASSAN II University Hospital, Fès 30000, Morocco; 2 Department of Medical Phisic, HASSAN II University Hospital, Fès 30000, Morocco

**Keywords:** cutaneous squamous cell carcinomas, facial localizations, radiation therapy

## Abstract

Cutaneous squamous cell carcinomas are very rare in children. The recommended treatment for localized cancers is surgery with sufficient margins which can sometimes be mutilating especially for facial localizations. We report a rare case of facial skin carcinoma in a 13-year-old girl measuring 3 cm in diameter infiltrating the tip of the nose. The treatment was an exclusive external radiation therapy with a dose of 70 Gy in 35 fractions in standard fractionation. The technique used was intensity-modulated conformational radiotherapy. It was proposed as an alternative to surgery which could be mutilating. A complete tumour response was obtained with a good aesthetic result and without major toxicity.

## Introduction

Cutaneous squamous cell carcinomas are very rare in children. In a study from the north of England, only six squamous cell carcinomas of the skin were seen in 28 years [1]. It is often associated with xeroderma pigmentosum (XP) [2]. It develops from the upper layers of the epidermis in the photo-exposed covex areas, and can progress at the lymph node and metastatic level [3]. The standard of treatment for localized cancers is surgery with sufficient margins which can sometimes be mutilating. Radiotherapy treatment is a good therapeutic alternative, especially for facial localizations [4]. We report the case of skin carcinoma of the nose in a 13-year-old girl treated with exclusive radiation therapy to prevent surgical mutilation.

## Observation

We report the case of a 13-year-old girl, without a pathological history, who consulted for skin symptomatology about an ulcerated lesion of the tip of the nose that gradually increased in volume for 5 months, treated by premedication without improvement.

**Figure 1.**
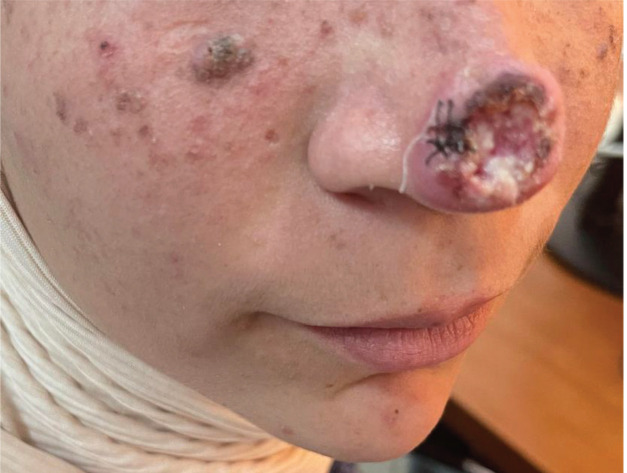
Ulcerous tumoural lesion of the tip of the nose of an infiltrating cutaneous squamous cell carcinomas.

She initially consulted a dermatologist who performed a complete clinical examination objectifying the presence of a tumoural lesion of the tip of the nose with central ulceration, meliceric crust, white area, and without polymorphic vascular structure. It measured 3 cm in diameter, uninfected and associated with multiple stable facial nevi suspecting a genetic variant of XP (Figure 1).

Histological examination of the tumoural biopsy shows that it is a well differentiated and infiltrating cutaneous squamous cell carcinomas. Given the patient’s young age, the clinical tumour size of 3 cm in this localization and the suspicion that she had a genetic variant of XP, it was preferred to proceed with an exhaustive imaging examination. A cervical**, facial and thoraco abdominal CT scan** showed limited tumour lesion at the tip of the nose without systemic or lymph node metastases, then the tumour was classified T2 N0 M0 Stage II with a high risk of recurrence.

An excisional surgery was initially proposed to the patient, but given her young age, the parents preferred another therapeutic alternative to minimize the risk of mutilation and its psychological repercussions. The case was then discussed in a multidisciplinary consultation meeting and the decision was made to opt for exclusive radiotherapy. Concurrent chemotherapy was not proposed because of the suspicion of a genetic variant of XP in the patient.

**Figure 2.**
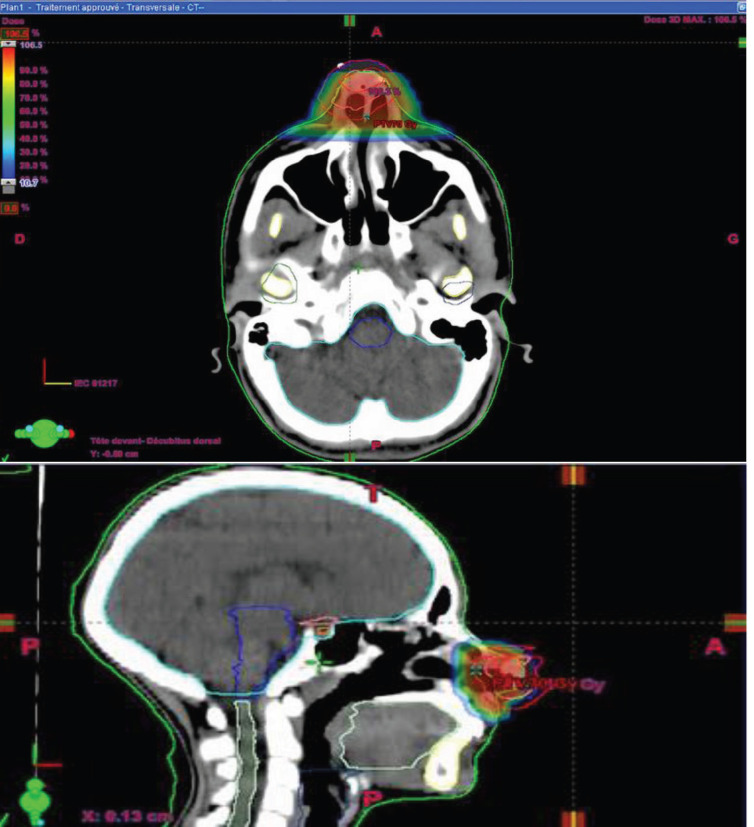
Radiation dose distribution at target volumes.

**Figure 3.**
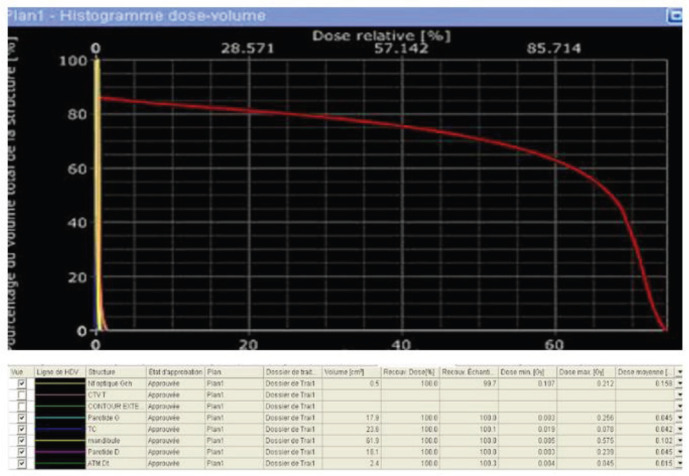
Histogram dose-volume.

For radiotherapy treatment, the dose received was 70 Gy in 35 fractions, 2 Gy per fraction, 5 days per week spread over 5 weeks with intensity-modulated conformational radiotherapy technique and using 6 MV high-energy X-photons. It allowed us to obtain good coverage of the tumour volume without using the bolus to increase the dose to the skin. For target volumes, the gross tumour volume (GTV) was the macroscopic tumour volume visible on the imaging, the clinical target volume (CTV) was a 5 cm margin on the GTV and the planning target volume (PTV) was a 5 cm margin on the CTV T. **No prophylactic lymph node’s radiotherapy as it is a superficial T2-classified tumour without positive lymph nodes.** The optimization of the dose to organs at risk was made for the eyes, optic nerves, crystallins, optic chiasma, brain trunk and spinal cord. The dosimetric constraints of all organs at risk were respected (Figures 2 and 3).

During the weekly and post-therapy monitoring, the only toxicity the patient had was a grade 1 NCI-CTCAE V5.0 radiodermatitis and was symptomatically treated with scar creams. Sun protection has also been prescribed.

**Figure 4.**
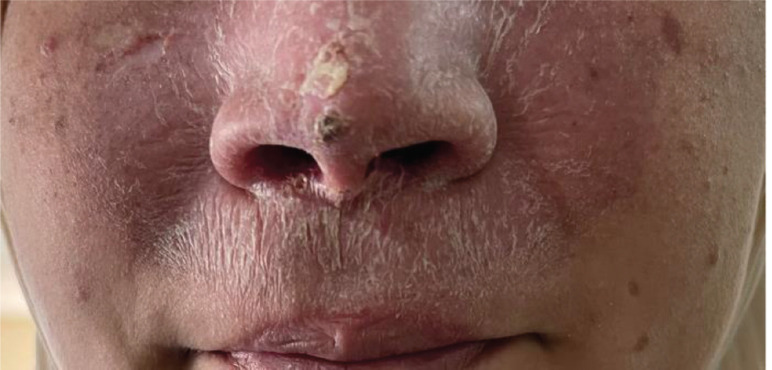
Evolution of the tumour after radiothérapy treatment.

The facial CT-scan performed after 6 months since the end of the radiotherapy showed a complete tumour remission with satisfactory clinical results (Figure 4). Given the risk of new lesions due to XP and exposure to ionizing radiation, close monitoring was planned with dermatologists to perform a biopsy in the slightest doubt.

## Discussion

Cutaneous squamous cell carcinomas develop with preference in photo-exposed regions, cervicofacial localization represents 75% of skin cancers [3]. It is exceptional in children and often associated with a genetic disease such as XP or its variants [5].

The treatment of skin carcinomas relies on surgical excision with sufficient margins of resection. In the absence of pejorative risk factors, specific survival after surgery is close to 100% at 3 years [6, 7].

Radiotherapy is an effective treatment allowing a high rate of complete response. It can be used in frail elderly, with significant comorbidity, as an alternative to mutilating surgery, in the case of locally advanced non-operable tumour or in addition to surgery in the case of derical factors of recurrence [8].

The surgical resection in our patient was going to be mutilating, taking more than half of her nose, and even the reconstruction was technically difficult. There is also the risk of recurrence or other lesions on the face, especially since it is a young girl with a high risk of genetic variant of XP.

In the literature, we found only rare cases of skin in children squamous cell carcinoma, none in this localization. The treatment performed in them was surgery and subsequent chemotherapy with adjuvant radiotherapy [10]. Our therapeutic attitude referred to that of the adult. To our knowledge, no similar treatment has been achieved in the child, which is both a strength and a limitation of our work.

The choice to opt for exclusive radiotherapy with a dose of 70 Gy was well justified [9], and it allowed us to give this child all the chances of remission without aesthetic repercussions that could have a significant psychological impact, especially since she is in the early adolescent phase [11].

Close surveillance with genetic consultation and optimal protection against photo ultraviolet (UV) exposure is necessary in order to detect and treat any suspicious lesion early [12].

## Conclusion

The management of skin carcinoma of the face in a child is particularly difficult because of its rarity. A multidisciplinary decision weighing the benefits and risks of each therapeutic modality is necessary for optimal treatment. What we can learn from our case report is the possibility of replacing surgery with exclusive radiotherapy in facial skin carcinomas to avoid mutilating surgery which impacts the quality of life of these young patients negatively.

## Conflicts of interest

The authors declare that they have no conflicts of interest.

## Funding

There was no funding for this work.

## Consent to participate

Parental and child consent was obtained.
